# Tumor-derived small extracellular vesicles promote breast cancer progression by upregulating PD-L1 expression in macrophages

**DOI:** 10.1186/s12935-023-02980-0

**Published:** 2023-07-14

**Authors:** Di Xu, Wen-Quan Chen, Ming-Xing Liang, Xiu Chen, Zhen Liu, Yin-Jiao Fei, Xin-Yi Shao, Yang Wu, Wei Zhang, Jin-Hai Tang

**Affiliations:** 1 Department of General Surgery, the First Affiliated Hospital of Nanjing Medical University, Nanjing, 210029 P.R. China; 2 The First Clinical college, Nanjing University of Chinese Medicine, Nanjing, 210029 P.R. China

**Keywords:** Breast cancer, Macrophages, Small extracellular vesicles, PD-L1, MicroRNA

## Abstract

**Background:**

The metastasis of breast cancer (BC) is a complex multi-step pathological process, strictly dependent on the intrinsic characteristics of BC cells and promoted by a predisposing microenvironment. Although immunotherapy has made important progress in metastasis BC, the heterogeneity of PD-L1 in tumor associated macrophages (TAMs) in BC and the underlying mechanisms in the metastasis development of BC are still not completely elucidated. Small extracellular vesicles (sEVs) represent essential interaction mediators between BC cells and TAMs. It is worth noting to explore the underlying mechanisms typical of sEVs and their role in the metastasis development of BC.

**Methods:**

The structure of sEVs was identified by TEM, while the particle size and amounts of sEVs were detected by BCA and NTA analysis. The specific PD-L1 + CD163 + TAM subpopulation in metastasis BC was identified by scRNA-seq data of GEO datasets and verified by IHC and IF. The function of TAMs and sEVs in metastasis BC was explored by RT-qPCR, WB, IF, flow cytometry and in vivo experiment. The expression profiles of plasma sEVs-miRNA in relation to BC metastasis was analyzed using next-generation sequencing. Further detailed mechanisms of sEVs in the metastasis development of BC were explored by bioinformatics analysis, RT-qPCR, WB and luciferase reporter assay.

**Results:**

In this study, we identified that the immunosuppressive molecule PD-L1 was more abundant in TAMs than in BC cells, and a specific PD-L1 + CD163 + TAM subpopulation was found to be associated with metastasis BC. Additionally, we found that BC cells-derived sEVs can upregulate the PD-L1 expression and induce the M2 polarization, enhancing the metastasis development both in vitro and in vivo. Also, Clinical data showed that sEV-miR-106b-5p and sEV-miR-18a-5p was in relation to BC metastasis development and poor prognosis of BC patients. Further mechanistic experiments revealed that BC-derived sEV-miR-106b-5p and sEV-miR-18a-5p could synergistically promoted the PD-L1 expression in M2 TAMs by modulating the PTEN/AKT and PIAS3/STAT3 pathways, resulting in the enhancement of the BC cells invasion and metastasis.

**Conclusions:**

Our study demonstrated that BC-derived sEVs can induce metastasis in BC through miR-106b-5p/PTEN/AKT/PD-L1 and miR-18a-5p/PIAS3/STAT3/PD-L1 pathways in TAMs. Therefore, the inhibition of these specific interactions of signaling pathways would represent a promising target for future therapeutic strategies for treatment of BC.

**Supplementary Information:**

The online version contains supplementary material available at 10.1186/s12935-023-02980-0.

## Introduction

Breast cancer (BC) is the most common malignant tumor affecting women and the second cause of mortality globally [1]. Although the commonly used treatment strategies allowed to make great progresses, the recurrence, metastasis, and drug resistance are still the most important challenges in cancer treatment [2, 3]. It is indeed reported that 20–30% of BC patients with metastasis at the initial diagnosis, and about 90% of related deaths are caused by metastasis, with the lungs being the most commonly affected organ [4]. The distant metastasis of BC is a complex multi-step process and the most accepted metastasis model is the “seed and soil” hypothesis proposed by *Stephen Paget*, which indicates that successful interaction of the intrinsic properties of tumor cells (seeds) and predisposing microenvironment (soil) for the development of metastasis [5]. Therefore, the only elimination of tumor cells may not be sufficient to effectively inhibit the progress of BC. Further, the heterogeneity of BC relies not only on tumor cells, but also on the interstitial cells in the tumor-associated microenvironments (TME) [6]. In the process of metastasis development, BC cells need to evade the immune system to survive [7], and so the investigation of the interactions between BC and immune cells, may be of great value to develop new immunotherapy strategies to treat BC.

Tumor-associated macrophages (TAMs), are important immune stromal cells accounting for 30-50% of immune cells, and they are in a dynamic transformation process of anti-tumor and tumor promotion. It was found that the increase of TAM and the decrease of M1/M2 TAM ratio in BC are associated with a poor prognosis [8, 9]. Recent studies demonstrated the complexity of TAM as the activation of macrophages in tumors does not follow the conventional M1/M2 activation model [10]. Macrophages were found to express different transcriptomes in response to different stimulation in vitro [11], however, how TAMs act in case of metastatic BC and their molecular mechanisms still remains to be elucidated.

Through the additional research about the correlation between TME and tumor progression, it was found that small extracellular vesicles (sEVs) play a crucial role in the intercellular communication between BC and metastasis microenvironment [12]. sEVs are bilayer membrane structures (30–150 nm), containing nucleic acid, protein, lipid, and other substances that play a role in the intercellular communication and signal transduction [13]. Among these cargos, microRNA (miRNA) is the most abundant in sEVs and it is implicated in the development of metastasis of multiple tumors [14]. Our previous studies identified the key roles of sEV-mediated delivery in the development of BC and drug resistance [15, 16]. However, the related underlying mechanisms are still not completely elucidated. Here, we therefore explored the sEVs-mediated communication between tumor and immune cells in the development of BC metastasis.

Programmed death ligand-1 (PD-L1) and its receptor programmed cell death 1 (PD-1) can suppress the killing effect of T cells, resulting in immune evasion of tumor cells [17, 18]. Immunotherapies targeting PD-L1 and PD-1 were often designed to cure PD-L1 positive tumors [19, 20]. However, some clinical studies have showed that PD-L1 and PD-1 blockers can also get meaningful effect in PD-L1 negative tumors [21, 22], suggesting a tumor and T cells-independent effect. Thus, some studies suggested that detecting the PD-L1 in immune cells rather than cancer cells, can screen suitable patients for using PD-L1 antibodies [23, 24].

In this study, for the first time, the alteration and functions of TAMs in metastasis BC were explored. Further detailed mechanistic experiments revealed that BC-derived sEV-miR-106b-5p and sEV-miR-18a-5p synergistically can promote the PD-L1 expression in M2 TAMs by regulating the PTEN/AKT and PIAS3/STAT3 pathways, contributing to BC metastasis, suggesting a new target for BC immunotherapy.

## Materials and methods

### Cell lines and culture conditions

MDA-MB-231 and THP-1 cells were purchased from Chinese Academy of Sciences and cultured in completed medium supplemented with 10% of Fetal Bovine Serum (FBS), 100 U/mL penicillin, and 0.1 mg/mL streptomycin at 37 °C with 5% CO2. Before conducting cell experiments, we replaced the culture medium with an antibiotic-free medium. During the cell culture process, we conducted mycoplasma detection by MycoBlue Mycoplasma Detector (Vazyme, Nanjing, China) to ensure that cells are free from mycoplasma contamination. Briefly, 1 µL cell culture medium supernatant was added with 24 µL MycoBlue buffer and 1 µL MycoBlue Enzyme. The mixture was incubated at 60 °C for 60 min and then detected by the color of reaction solution, compared with positive controls.

CD14 + monocytes were isolated from human peripheral blood mononuclear cell (PBMC) through bead separation technology. The macrophages were induced by stimulation of CD14 + monocyte with 50 ng/mL M-CSF or THP-1 with 100 ng/mL of PMA and they were cultured in RPMI-1640 with 10% of FBS at 37 °C with 5% CO2.

### Co-culture treatment

To analyze the different cells interaction influence, a co-culture system was established using transwell chambers (0.4 μm, Corning, New York, USA). MDA-MB-231 was placed in the lower chamber while macrophages were put in the upper chamber. The mechanism is based on the fact that the smaller polycarbonate membrane guarantee cells are unable to migrate into each other contrarily to sEVs.

### Blood and tissue samples collection of patients

Plasma samples of healthy volunteers, patients with early BC (Stage I) and patients with advanced BC were obtained from patients affected by BC from the Jiangsu province hospital. All tissue samples and corresponding paracancerous tissues are collected from patients affected by BC during operation or puncture from the Jiangsu province hospital. All patients were admitted for the first time and had not received any treatment before. The clinical pathological diagnosis was confirmed by at least two pathologists and the informed consent forms were signed before enrollment. The current study and the inclusion criteria adopted were approved by the Ethics Committee of Jiangsu province hospital (2020-SR-457). The clinical characteristics of patients were shown in Supplementary Table 1.

### Immunohistochemistry staining (IHC)

Tissue samples were embedded in paraffin, cut into 4 μm slices and mounted on glass slides. Following the baking at 60℃ for 2 h, slices were put in a gradient alcohol for the dehydration treatment. The expression of CD68 and CD163 was detected by an IHC commercial kit (#KIT-9710, MXB, Fujian, China). DAB Kit (#DAB-2031, MXB, Fujian, China) was applied to show the staining results and mixed according to manufacturer’s instructions to expose staining difference. IHC scores were determined using the light microscopy (AX10, Zeiss, Germany), calculated as the staining percentage scores, and multiplied by the staining intensity scores described as our previous study. The primary Abs used are listed in Supplementary Table 2.

### Immunofluorescence (IF)

The cells were put on the cell crawl sheet, and for tissue samples, they were fixed using 4% of formaldehyde. The 0.1% NP40 was used to permeate the cell membrane, followed by 5% BSA which was used for blocking for half an hour. The primary Ab was added and incubated at 4° overnight. Next, the secondary fluorescent Ab was added and incubated for 1 h. Finally, antifluorescence quenching agent containing DAPI was added to slice. Samples were observed using fluorescence microscope (Thunder Image, LEICA, German).

### Flow cytometry

To detect macrophage markers, cells were labelled with CD68-PE and CD163-PE/CY7 human Abs (Biolegend, San Diego, California, USA). Briefly, pre-treated macrophages were digested using PBS with 2.5 mM EDTA and stopped using PBS with 0.5% BSA. The precipitate was then resuspended using 100 µl of PBS, added with 5 µl of human FcR Blocking Agent (Biolegend, San Diego, California, USA), and finally mixed and incubated for 10 min to block the Fc receptor. After washed, cells were resuspended in 300 µl of PBS with 3 µl CD163-PC7 to stain cell surface Ab for 30 min. Then, the rupture of cells membranes was induced by fixation/permeabilization for 45 min. After PBS washing, cells were resuspended in 300 µl PBS with 3 µl of CD68-PE to stain cell intracellular Ab for 30 min. The percentage of CD68/CD163 macrophage cells was quantified by using Cytoflex flow cytometer (Beckman, Brea, California, USA).

### Bioinformatics analysis

The correlation between the level of PD-L1 and miRNAs and overall survival (OS) in BC patients was analyzed using the Kaplan–Meier plotter online tool (http://kmplot.com/analysis/) based on TCGA database. The expression of PD-L1 and miRNA in BC of TCGA database was analyzed using UALCAN online tool (http://ualcan.path.uab.edu). The correlation between macrophage infiltration and OS in BC patients was analyzed using the TIMER2.0 online tool (http://timer.cistrome.org). The correlation between PD-L1 expression and macrophage infiltration in BC was also analyzed using the TIMER2.0 online tool. The single-cell expression and spatial localization of PD-L1 in BC of GEO database were analyzed using the TISCH online tool (http://tisch.comp-genomics.org/documentation/). The correlation between miRNA and mRNA expression in breast cancer were analyzed using the ENCORI online tool (https://rnasysu.com/encori/agoClipRNA.php?source=mRNA) based on TCGA database.

### In vivo experiment

Animal experiments were approved by the Institutional Animal Care and Use Committee of NMU (IACUC-2,203,065). Five weeks-old female BALB/c nude mice were purchased from Vitalriver (Beijing, China). Luciferase-labeled MDA-MB-231 BC cells and THP-1 derived macrophage cells were suspended or mixed in PBS with the cell density reaching 1.0 × 10^6^ cells/100 µL, and 100 µL of the cell suspension was injected into the tail vein of mice to establish a transfer model. The body weight of mice was regularly monitored. Then, imaging system (IVIS Spectrum, PerkinElmer, Waltham, USA) was used to assess the fluorescence signals and tumor bearing of mice, using 20 mg/mL of D-Luciferin potassium salt (Yeasen, Shanghai, China). All mice were euthanized and lung were collected. The lungs of mice were isolated, flushed with physiological saline for 5 min and fixed with Bouin’s solution for 24 h. The metastatic pulmonary nodules on lung surface were then counted using an anatomical microscope.

To confirm the role of sEVs in BC metastasis, 100 µL of mixed MDA-MB-231 BC cells and THP-1 derived macrophage cells suspension (5.0 × 106 cells/100 µL) were injected into the breast pads of mice to establish orthotopic xenografts models. Body weight and the volume of the tumor were measured. The tumor volume was calculated as a×b2/2, where “a” and “b” are the largest and smallest diameters, respectively. When the tumor volume reached 50mm3, the Group A mice were injected with 100 µg/100 µL sEVs derived by BC cells, while the Group B mice were injected with 100 µL PBS every 3 days for 5 times. H&E staining of the main organs was performed to investigate the location of the metastasis.

### Isolation, verification and labeling of sEVs

sEVs were extracted by sequential ultracentrifugation: briefly, cells culture was replaced by medium containing 10% of EV‑depleted FBS. Then, cell culture medium was collected and centrifuged at 300 × g for 10 min, 2000 × g for 15 min, and finally at 12,000 × g for 30 min to remove cell debris. After filtration through 0.22-µm filters, the supernatant was ultracentrifuged at 100,000 × g (Beckman Type 90 Ti) for 2 h. The sEVs pellets were washed in PBS and ultracentrifuged at 100,000 × g for 2 h again. All samples were re-suspended in PBS for further analyses.

The isolated sEVs were absorbed by carbon coated copper networks for 2 min, washed in ddH2O, and finally negative staining with uranium acetate for 2 min. All samples were observed by Transmission electron microscopy (TEM) (JEM-1010, JEOL, Japan). The size distributions and concentrations of diluted sEVs were analyzed by Nanoparticle Tracking Analyzer (ZetaView®PMX120, Particle Metrix, German).

sEVs were labeled with a PKH26 Labeling Kit (Sigma-Aldrich, Saint Louis, USA) following the manufacturer’s instructions. The uptake of PKH26-labeled sEVs by macrophages was examined using confocal microscopy (Stellaris STED, LEICA, Germany).

### Total protein extraction and Western blot (WB)

Cell lysis buffer plus with phenylmethanesulfonyl fluoride (PMSF) diluted 1:100, diluted 100x protease inhibitor cocktail and diluted 50x phosphatase inhibitor cocktail A (Beyotime, Shanghai, China) were used to extract total cells and isolated sEVs proteins. The proteins extraction process, protein quantification, Western blotting (WB) analyses and proteins visualization were described as previously study [25]. The primary Abs used were listed in Supplementary Table 2.

### Total RNA extraction and real-time polymerase chain reaction (RT-qPCR)

Total RNA was extracted from cells using a total RNA Kit (Tian Gen, Beijing, China), while total RNA from sEVs was extracted using a Trizol ls Reagent (Thermo Fisher, Waltham, USA) following the manufacturer’s instructions. cDNA was synthesized using miRNA plustail method (Sangon, Shanghai, China), miRNA stem-loop method (Sangon, Shanghai, China) and HiScript II Q RT SuperMix (Vazyme, Nanjing, China), respectively. Gene expression was examined by RT-qPCR using the ChamQ SYBR qPCR Master Mix (high ROX premixed) (Vazyme, Nanjing, China) and a StepOnePlus Real-Time PCR System (Thermo Fisher Scientific, Waltham, Massachusetts, USA). The PCR run method of the manufacturer’s instructions was used. The Ct values were normalized to U6 or GAPDH genes, used as extraction control. All primers sequences are reported in Supplementary Tables 3–4.

### Next-generation sequencing of plasma sEVs

The expression profiles of sEVs-miRNA were investigated by RNA-seq Illumina Hiseq2500 (Illumina, San Diego, California, USA) at Biomedical public service platform (Nanjing, China).

### Luciferase reporter assay

The 3’-untranslated (UTR) region of PTEN or PIAS3 targeted by miR-106b-5p and miR-18a-5p were predicted by Targetscan online tool (https://www.targetscan.org/vert_80/) and Starbase online tool (https://starbase.sysu.edu.cn/). The wild-type (WT) or mutant (MUT) reporter plasmids were constructed (Ribobio, Guangzhou, China) and a co-transfection was performed with miR-106-5p and miR-18a-5p mimics into macrophages when cells reached a confluence of 70%. After 48 h, cell lysis was used to split and 20 µL cell lysate was added to dual-luciferase reporter assay system (Yeasen, Shanghai, China), measuring the reporter gene activity.

### Cell transfection

Cells were transfected with negative control (NC), predesigned miRNA minics, or predesigned si-PTEN and si-PIAS3 using Lipo8000 transfection reagent (Beyotime, Shanghai, China). Briefly, cells were planked with the density at transfection was 40% in advance. Then, prepare two tubes of mixed reagent. A: 250 µL Opti-MEM + 100 pmol miRNA minics/siRNA; B: 250 µL Opti-MEM + 4 µL Lipo8000. After 5 min, we mixed two tubes and co-cultured for 20 min. Finally, the compound was added to each well for 48 h before further examination.

### Statistical analysis

All experiments were conducted in triplicates. Data were analyzed using t-test for independent samples between two groups, and using one-way ANOVAs between multiple groups. A P-value < 0.05 was considered statistically significant.

## Results

### Immunity alteration of TAMs in the metastasis BC

**Fig. 1:**
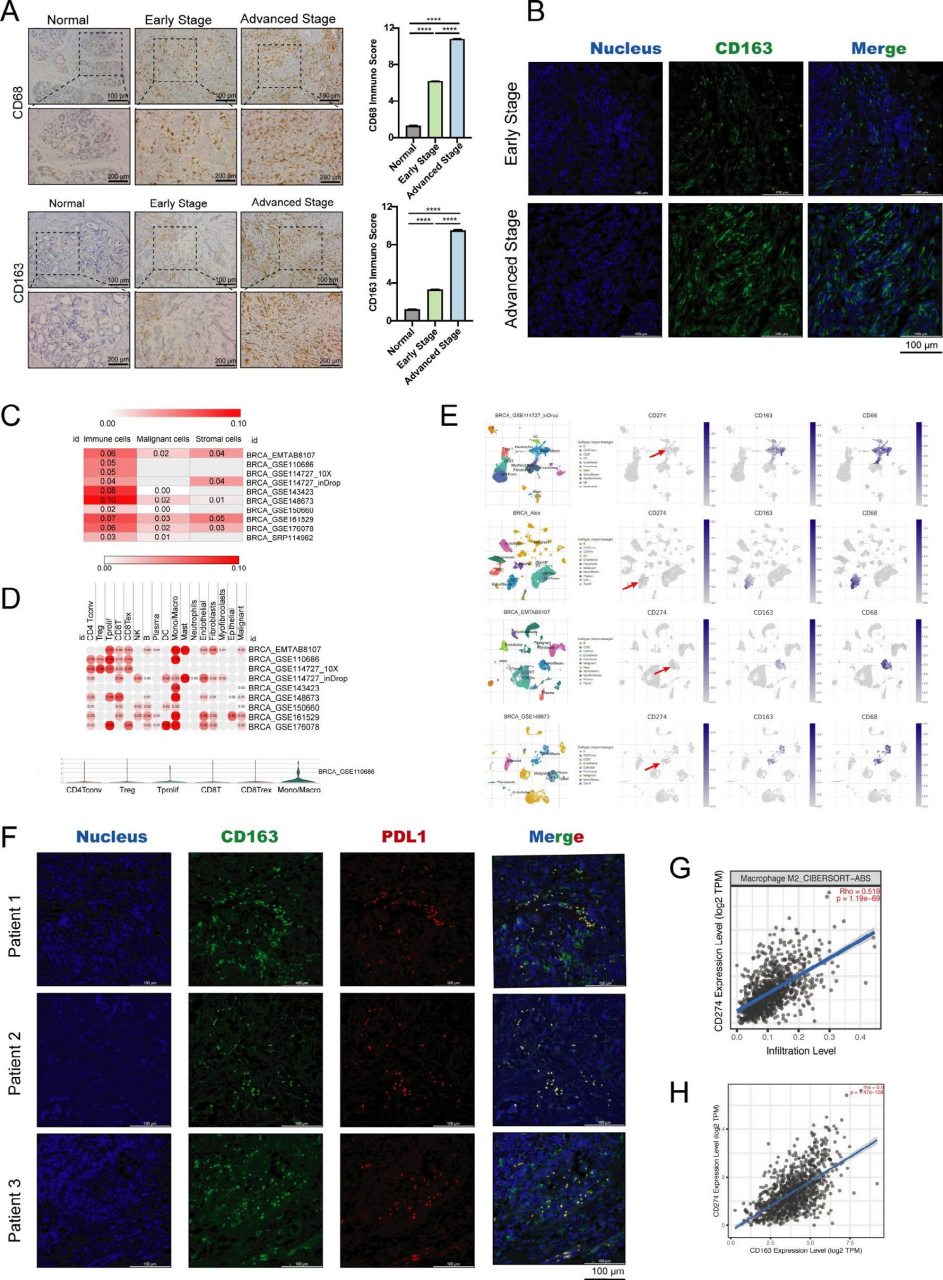
Alteration of the immunity of TAMs in BC metastasis. (**A**) The CD68 and CD163 expression levels of TAMs in early BC tissues, advanced BC tissues, and the related para cancerous tissues. (**B**) The proportion of CD163 + TAMs in early and advanced BC tissues detected by IF staining. (**C**) Heat map showing the PD-L1 expression between BC cells, immune cells, and stromal cells based on scRNA-seq data from GEO datasets. (**D**) Heat map showing the PD-L1 expression in different types of immune cells. (**E**) The distribution of PD-L1 in different cell subsets based on scRNA-seq data from GEO datasets. (**F**) Co-localization of PD-L1 and CD163 on TAM in BC observed by IF. (**G**) The correlation between M2 macrophage infiltration and PD-L1 expression analyzed using CIBERSORT-ABS method. (**H**) The correlation between PD-L1 and CD163 expressions

TAMs were commonly reported as major immuno-suppressor cell types in the TME of BC [26]. It was found that more M2 macrophages infiltrate, the worse the OS rate of BC patients (Supplementary Fig. 1A-B). However, the activation of TAMs in metastasis BC has not been fully investigated, and it was suggested that it may not follow the standard M1/M2 activation model [10]. First, we detected the infiltration and immune changes of TAMs in early BC tissues, advanced BC tissues and in the corresponding paracancerous tissues by IHC and IF. We found that the macrophages infiltrating was significantly more in tumor tissue than that in para-cancer tissue. In addition, the proportion of CD163 + M2 in advanced BC was significantly higher than in early BC, indicating that M2 cells are potentially key cells for the development of metastasis of BC cells (Fig. 1A). Also, IF results confirmed the increased proportion of CD163 + M2 cells in advanced BC than in early BC (Fig. 1B). All together, these findings suggested that more M2 cells can be recruited during the process of tumor occurrence and metastasis.

Given the critical function of macrophages in the regulation of the immune response, the correlation between TAM and immune checkpoint ligands in BC was analyzed using TCGA and GEO databases. Interestingly, the expression of PD-L1 (CD274) between normal and BC tissues had no statistically significant difference, and it was not significantly associated with the OS rate of BC patients (Supplementary Fig. 1C-E). Therefore, we speculated that PD-L1 is more related to immune cells than tumor cells in BC. To further investigate the expression of PD-L1 in BC TME, scRNA-seq data of GEO datasets were analyzed to map TAMs in BC and we discovered that PD-L1 was significantly more abundant in immune cells than tumor and stromal cells in BC (Fig. 1C). It was found that PD-L1 expressed most significantly in macrophages (Fig. 1D), and especially in CD163 + macrophage subgroup (Fig. 1E). Thus, IF was used to confirm the co-location between PD-L1 and CD163, founding that PD-L1 was strongly expressed on macrophages and located on CD163 + M2 in advanced BC (Fig. 1F). Further, the correlation analysis showed that PD-L1 was significantly increased in M2 type TAM and CD163 in BC (Fig. 1G-H). Together, these data indicated a novel PD-L1 + CD163 + TAMs subgroup within the BC TME.

### BC cells induce macrophages to promote metastasis in vivo

**Fig. 2:**
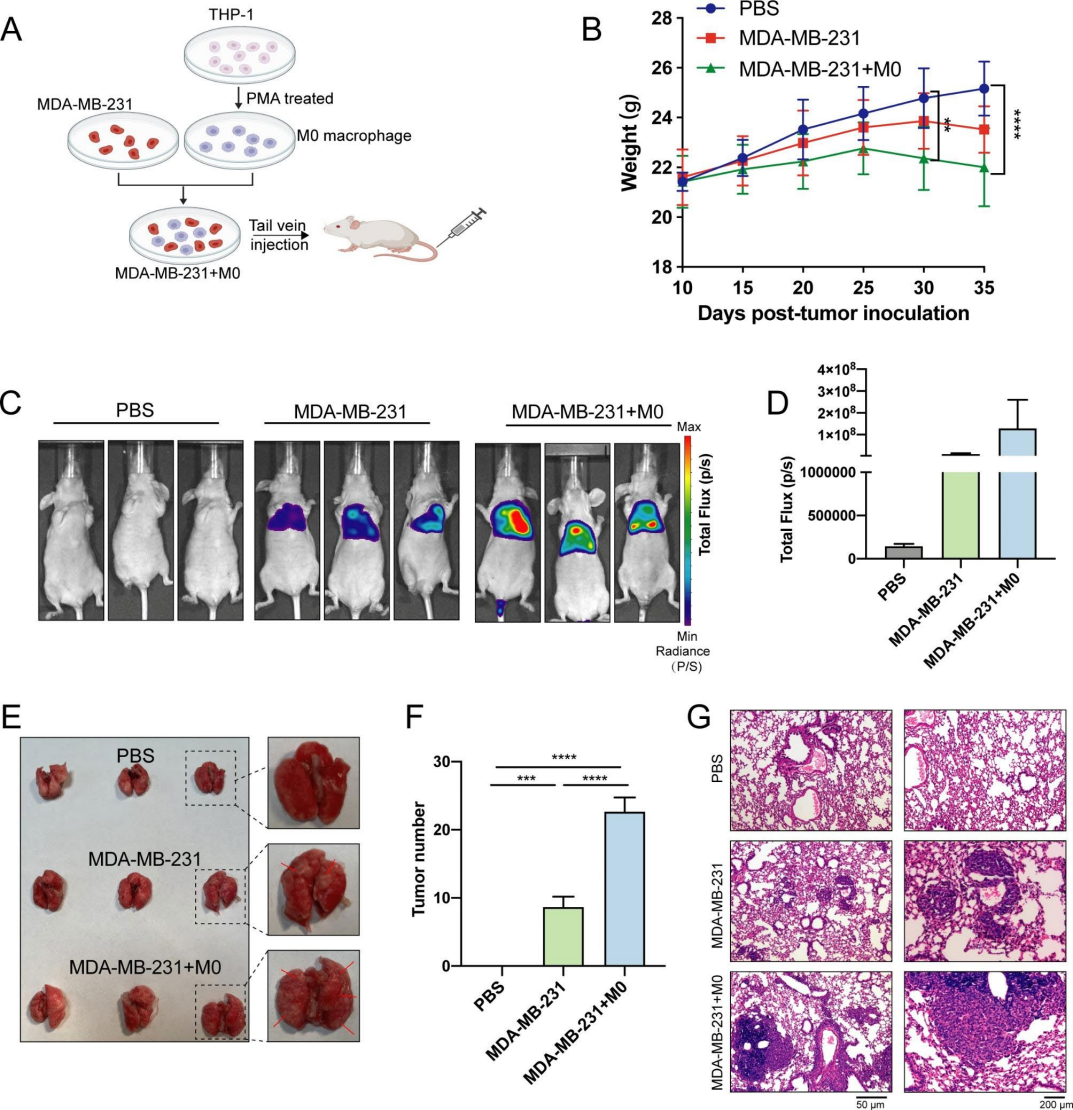
BC cells inducing M2 type differentiation in vivo to promote metastasis. (**A**) Schematic diagram of the in vivo experiments. (**B**) The body weight changes of mice after tail vein injection. (**C-D**) In vivo imaging and total flux of mice showing the load of tumor cells. (**E**) Representative images of lung metastasis in mice. (**F**) Quantification of lung metastasis in mice. (**G**) The characteristics of lung metastasis in mice analyzed by H&E staining

To investigate the function of macrophages in BC metastasis, MDA-MB-231 and M0 cells were separately or jointly injected into tail vein of mice (Fig. 2A). The body weight of mice was regularly monitored, with an earlier body weight loss in the MDA-MB-231 and M0 mixed-injection group (Fig. 2B). Bioluminescent imaging showed indeed that the mixed injection significantly aggravated the metastasis of the lungs (Fig. 2C, D). The images and quantification of lung metastasis also confirmed a significant increased number of lung metastases in this group (Fig. 2E, F), as well as H&E staining in which the size of the metastasis was found to be greater in the same group (Fig. 2G). The above in vivo experimental data showed that M0 were involved in the process of BC metastasis, which can be also stimulated by BC cells that determined the polarization into the M2 phenotype.

### BC cells promote the polarization of M2 macrophages and PD-L1 expression in vitro

**Fig. 3:**
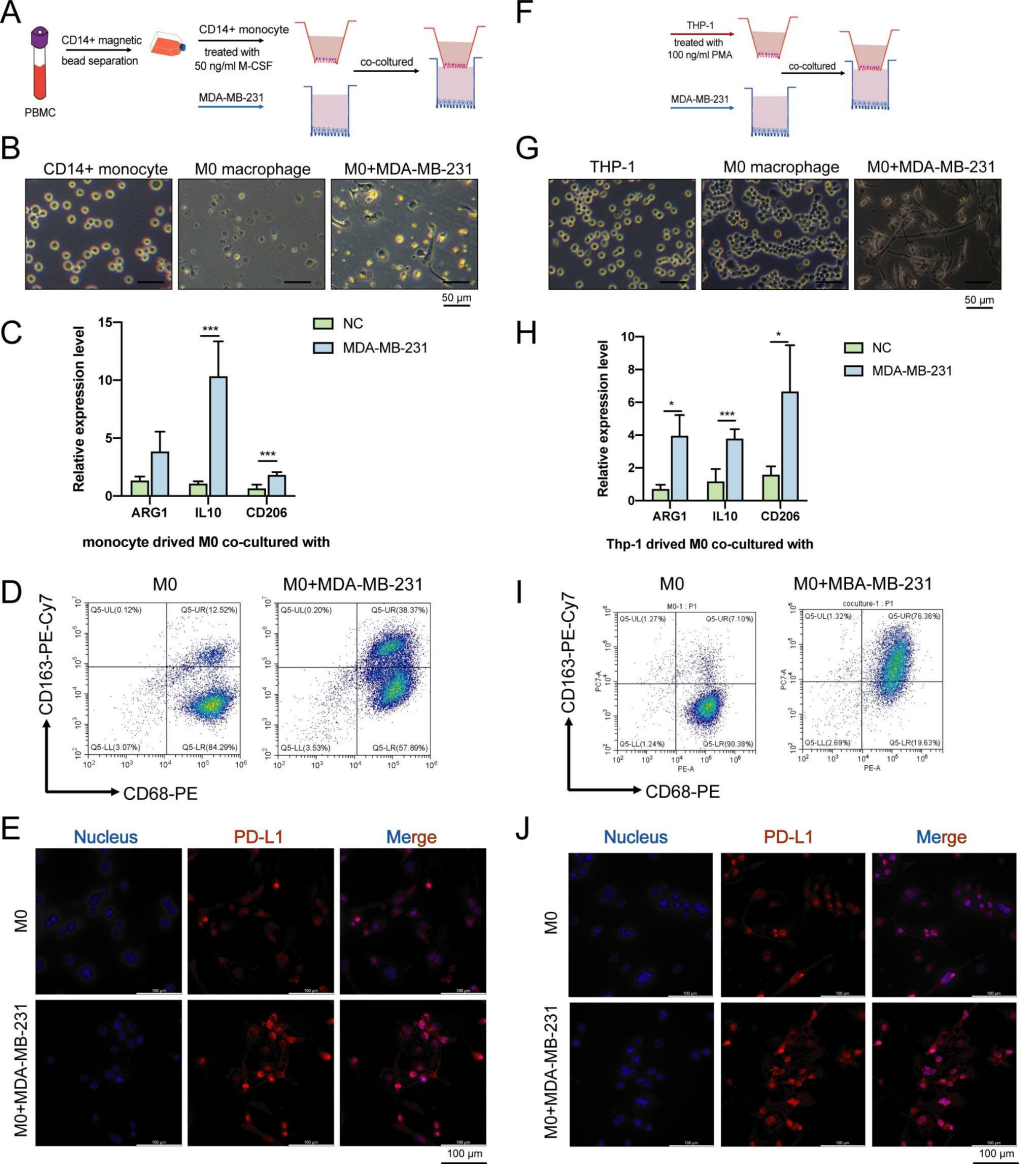
BC cells promoting the polarization of M2 macrophages and PD-L1 expression in vitro. (**A**) Co-culture schematic diagram between macrophages derived from CD14 + monocytes and BC cells MDA-MB-231. (**B**) Representative optical microscope representations of CD14 + monocytes, induced M0 macrophages, and macrophages after 48 h of co-culture. (**C**) The expression of M2 markers (CD206, ARG1 and IL-10) detected by RT-qPCR in CD14 + monocytes derived M0 after co-culture. (**D**) The proportion of CD163 + CD68 + macrophages after co-culture analyzed by flow cytometry. (**E**) The PD-L1 expression of CD14 + monocytes derived M0 after co-culture detected by IF. (**F**) Co-culture schematic diagram between THP-1-derived M0 and MDA-MB-231 cells. (**G**) Representative optical microscope pictures of THP-1 cells induced M0, and macrophages after 48 h of co-culture. (**H**) The expression of M2 markers (CD206, ARG1 and IL-10) detected by RT-qPCR. (**I**) The proportion of CD163 + CD68 + macrophages after co-culture which was analyzed by flow cytometry. (**J**) The PD-L1 expression detected after co-culture by IF.

To prove the polarization of macrophages when infiltrating tumor cells in vitro, the PBMC were extracted from human cells, then separated by CD14 + magnetic bead and turned into M0 by M-CSF (Fig. 3A). After co-culture with highly metastatic BC cells MDA-MB-231, the morphological analysis found that the cells were irregular and with long pseudopods, which was significantly associated with M2 type macrophages (Fig. 3B). Also, the expression level of M2 markers (CD206, ARG1 and IL-10) in the co-culture group were significantly upregulated detected by RT-qPCR (Fig. 3C). Flow cytometry showed that CD163% in the co-culture group increased significantly (Fig. 3D). Additionally, the PD-L1 expression in the nucleus of macrophages was found to be increased by IF in the co-culture group (Fig. 3E). At the same time, we co-cultured THP-1 derived M0 with MDA-MB-231. Morphology, RT-qPCR, and flow cytometry analysis all evidenced the M2 polarization induced by BC cells (Fig. 3F-J).

### sEVs from BC cells promote M2 polarization and PD-L1 expression in macrophages

**Fig. 4:**
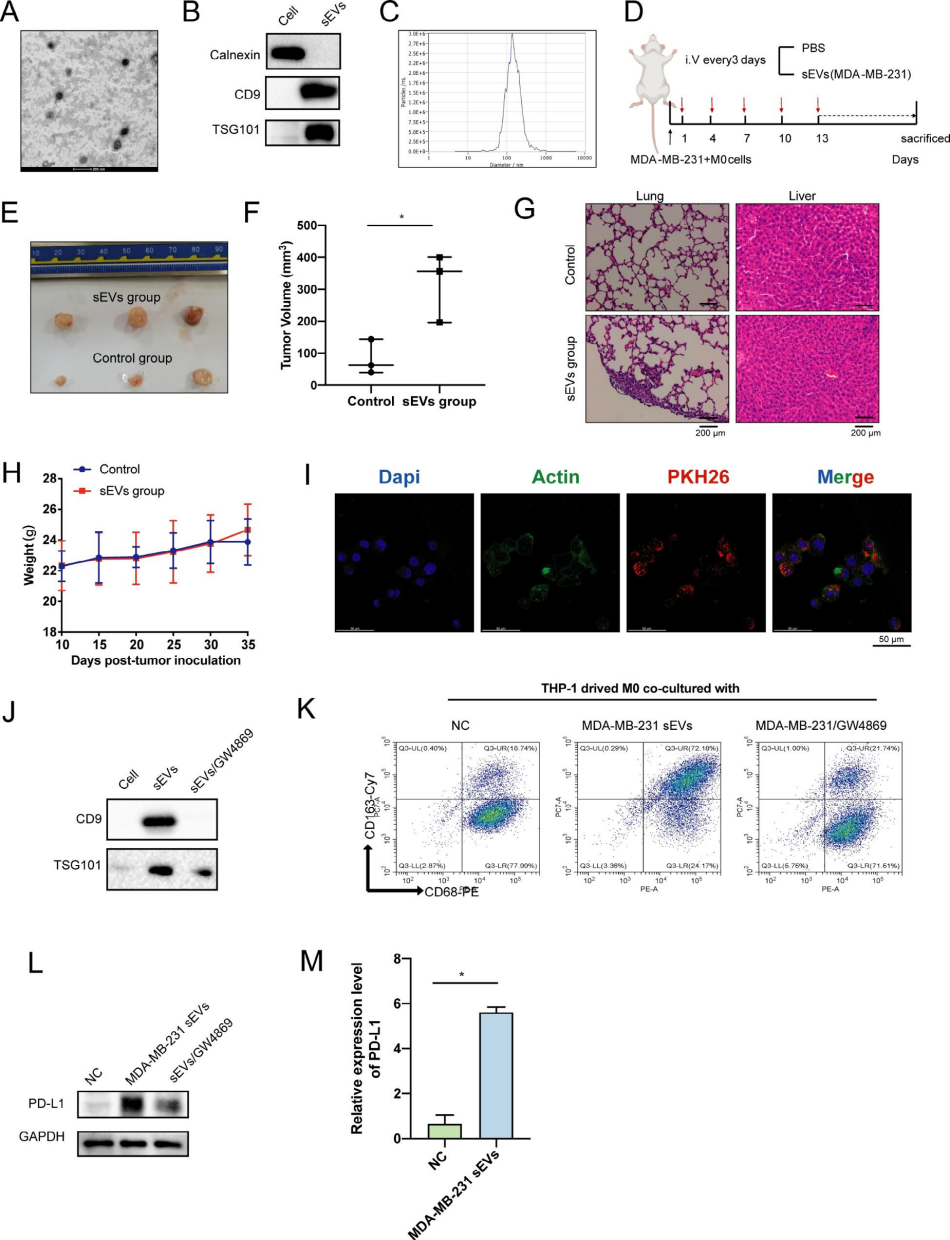
sEVs from BC cells promoted the PD-L1 expression in M2 cells. (**A**) Identification of the structure of sEVs isolated from MDA-MB-231 cells observed by TEM; scale bar = 200 nm. (**B**) Identification of sEVs marker proteins by WB. (**C**) Size analysis of sEVs by NTA. (**D**) Schematic diagram of the in vivo experiments. (**E**) Pictures of dissected tumors of mice. (**F**) Related sizes of dissected tumors of mice. (**G**) H&E staining of lung and liver of mice. (**H**) Weight growth curves of mice during the injection of sEVs. (**I**) IF detection of phagocytosis of BC cell derived sEVs by macrophages. (**J**) Inhibition of sEVs secretion by GW4869 observed by WB. (**K**) Proportion of CD163 + CD68 + macrophages co-cultured with BC derived sEVs detected by flow cytometry. (**L-M**) Protein and mRNA level of PD-L1 in macrophages after co-culture with BC derived sEVs.

Therefore, we started with exploring how BC cells can mediate the M2 polarization of macrophages. sEVs have been identified as essential mediators of the crosstalk between BC cells and TAMs, that is responsible for transferring RNA molecules from donor to recipient cells. Compared with normal cells, tumor cells release more secretions due to the more active metabolism. The sEVs extracted from the supernatant of BC cells were verified by TEM, NanoSight tracking, and WB analyses (Fig. 4A-C). Here, we found that BC cells-derived sEVs can significantly promote tumor growth and metastasis in vivo, suggesting the essential role of sEVs for the development of BC (Fig. 4D-F). Also, we found that the metastasis sites were mainly in the lung, while no metastasis were found in the liver (Fig. 4G). No statistically significant difference in the BW of mice during the injection of sEVs was found, suggesting the absence of toxicity (Fig. 4H).

Considering the important roles of sEVs and TAMs in BC metastasis, we hypothesized that TAMs may be modulated by BC-derived sEVs. The IF staining showed indeed that BC-derived sEVs could be effectively absorbed by macrophages (Fig. 4I), and the finding that GW4869 act as an inhibitor of the sEVs secretion synthesis and release [27],was further confirmed by our WB results (Fig. 4J). We found that when co-cultured with MDA-MB-231 cells, the proportion of M2 cells increased, while the addition of GW4869 was able to effectively inhibit the M2 differentiation of macrophages (Fig. 4K). Importantly, we found that the mRNA and protein levels of PD-L1 simultaneously significantly up-regulated in macrophages co-cultured with MDA-MB-231 sEVs by WB and RT-qPCR analysis (Fig. 4L-M). Therefore, BC cells may promote the M2 differentiation and PD-L1 expression in macrophages through sEVs-mediated mechanisms.

### Screening miRNAs enriched in plasma sEVs of metastatic BC

**Fig. 5:**
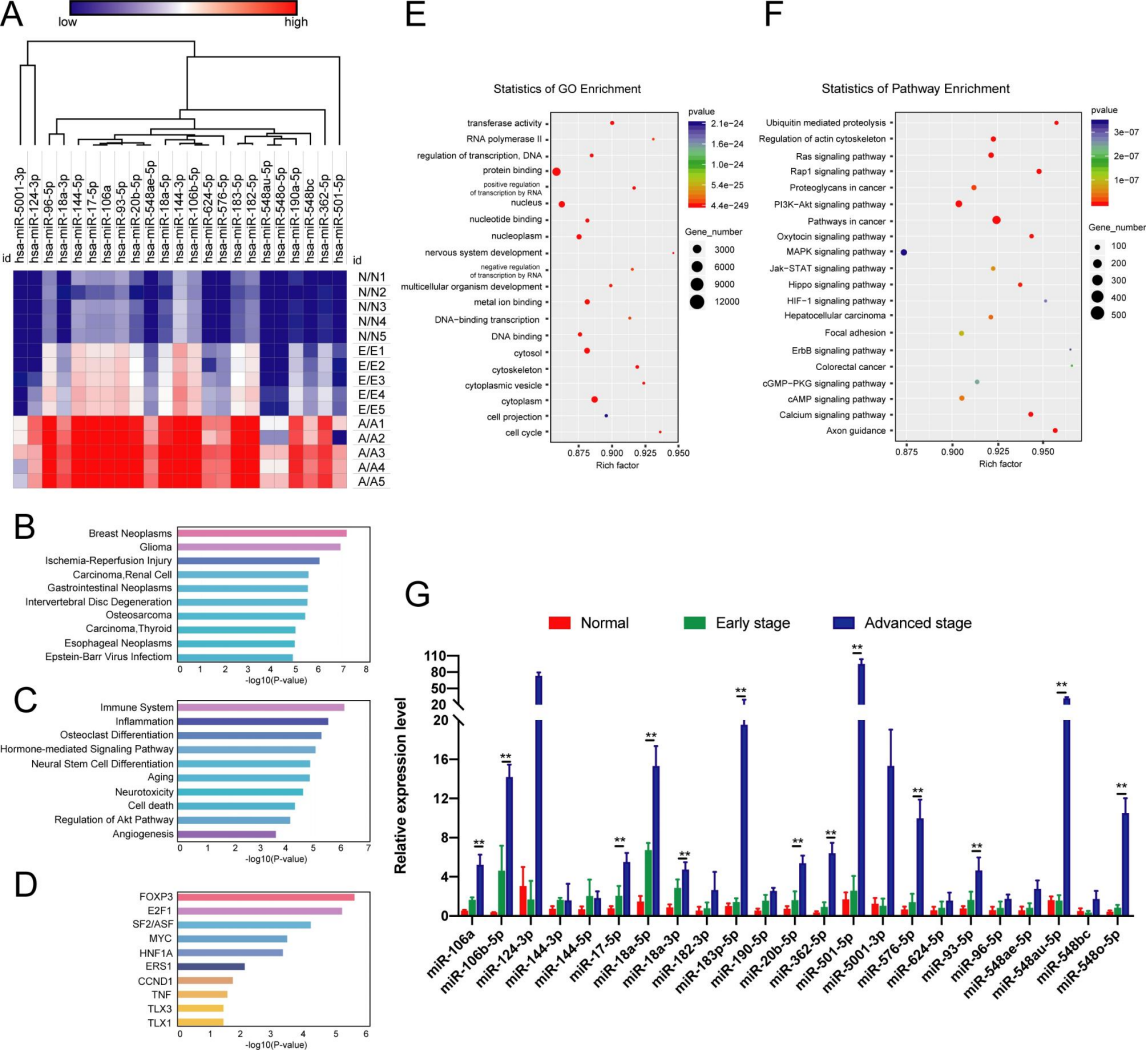
Screening miRNAs in enriched plasma sEVs of metastatic BC patients. (**A**) Heat map of differential expression of miRNAs among group N, group E and group A. (**B-D**) Disease enrichment analysis, functional enrichment analysis, and target genes analysis of differential expression miRNA among the three groups. (**E-F**) GO functional annotation analysis and KEGG pathway analysis of differential expression miRNA target genes among the three groups. (**G**) The differential expression of miRNAs in plasma sEVs among the three groups verified by RT-qPCR.

MiRNAs have been found to be the most abundant cargos in sEVs. To find the relationship between miRNAs and metastasis, 5 healthy volunteers, 5 patients with early BC (Stage I) and 5 patients with advanced BC (Stage IV) were selected for plasma sEV-miRNA sequencing. No significant difference among different groups were found regarding the age, however, significant differences were found in relation to the tumor size, lymph node metastasis, and ki67 expression (Supplementary Table 1). A total of 15 volunteers or patients from each group were also involved to verify the sequencing results. A total of 42 differentially up-regulated miRNAs expressions according to the difference multiple of 2 were found between each group, and they were higher in the metastasis group (A) > early group (E) > normal group (N) (Fig. 5A). The enrichment analysis showed that differential sEVs-miRNAs were significantly associated with BC, confirming also the tumor specificity of secreted sEVs-miRNAs (Fig. 5B). In addition, functional enrichment analysis showed that differential sEVs-miRNAs were significantly correlated with immune and inflammatory systems, and target gene enrichment analysis showed that differential sEVs-miRNAs were significantly correlated with the immune-related molecules FOXP3, E2F1 and so on (Fig. 5C-D). These findings indicated that these sEVs-miRNAs represent important regulatory factors that connect tumor and immune cells in TME.

Target genes of differentially expressed miRNAs were predicted by TargetScan and Starbase databases, then GO function annotation and KEGG pathway enrichment analyses of the predicted target genes were performed to explore the related biological functions. GO analysis proved the role of miRNAs in many biological processes, including signal transduction and transcriptional regulation, as well as protein binding, metal ion binding and DNA binding properties (Fig. 5E). On the other side, KEGG analysis demonstrated that cancer-related pathways, like PI3K-AKT and Ras signal pathways, etc. were significantly enriched (Fig. 5F). Therefore, 5 plasma samples from each group were collected and the related sEVs-miRNA expression was evaluated by RT-qPCR and TCGA database, confirming that the expressions of miR-106b-5p, miR-17-5p, miR-18a-3p, miR-18a-5p, miR-362-5p, miR-501-5p, miR-548ae-5p, miR-548o-5p, miR-576-5p, miR-624-5p, and miR-93-5p were significantly higher in plasma sEVs of patients with advanced BC than those of patients with early BC (Fig. 5G). Also, based on the TCGA database, the expression of these miRNAs was also significantly higher in BC tissues than in adjacent tissues, and it was associated with a poor prognosis in BC patients (Supplementary Fig. 2A-B).

### BC-derived sEV-miR-106b-5p and sEV-miR-18a-5p induce PD-L1 expression in macrophages and regulate their polarization

**Fig. 6:**
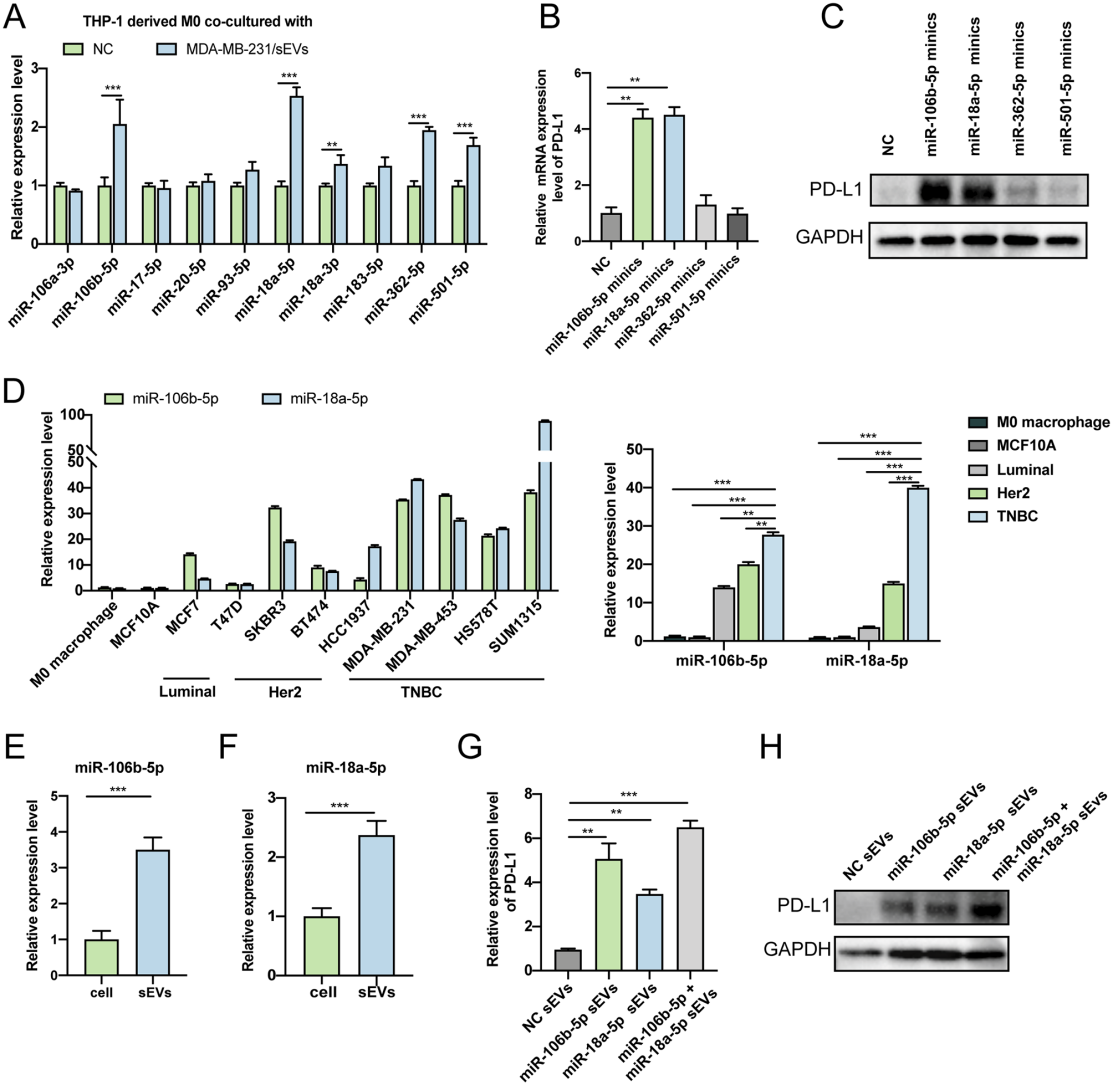
BC-derived sEV-miR-106b-5p and sEV-miR-18a-5p induce PD-L1 expression in macrophages and regulate their polarization. (**A**) The expression of the top 10 upregulated miRNAs in the macrophages co-cultured with sEVs from BC cells using a RT-qPCR assay. (**B-C**) The mRNA and protein levels of PD-L1 transfected with miR-106b-5p, miR-18a-5p, miR-501-5p and miR-362-5p mimics in macrophages. (**D**) The expression differences of miR-106b-5p and miR-18a-5p between M0 macrophages, normal breast cells and BC cells. (**E-F**) The expression differences of miR-106b-5p and miR-18a-5p between cells and cell derived sEVs. (**G-H**) The mRNA and protein levels of PD-L1 co-cultured with high levels miR-106b-5p and/or miR-18a-5p sEV.

To identify which sEVs-miRNAs are involved in the regulation of M2 polarization and related PD-L1 expression, we investigated the top 10 upregulated miRNAs by RT-qPCRs and confirmed that miR-106b-5p, miR-18a-5p, miR-501-5p and miR-362-5p were significantly upregulated in macrophages when co-cultured with sEVs from BC cells (Fig. 6A). Then, the mimics of four identified miRNAs (miR-106b-5p, miR-18a-5p, miR-501-5p, and miR-362-5p) were transfected into macrophages in separate experiments (Supplementary Fig. 3A). We found that mRNA and proteins levels of PD-L1 were increased by miR-106b-5p and miR-18a-5p significantly (Fig. 6B-C), while under the RNase A treatment, the levels of miR-106b-5p and miR-18a-5p in MDA-MB-231 cell culture medium were not degraded. However, following the combined treatment with Triton X-100, RNase A was able to effectively trigger the degradation of miR-106b-5p and miR-18a-5p, indicating that the extracellular secretions of miR-106b-5p and miR-18a-5p are not exposed to the medium, but protected by the membrane (Supplementary Fig. 3B-C).

Subsequently, we compared the expression of miR-106b-5p and miR-18a-5p between BC cells and macrophages. miR-106b-5p and miR-18a-5p were found significantly higher in BC cells than in M0 and normal cells (Fig. 6D). The expression analysis in different types of BC showed that miR-106b-5p and miR-18a-5p were the most abundant in the TNBC type. Although TNBC breast cancer only accounts for about 20% of patients enrolled, it is associated with the worst prognosis [28, 29]. Indeed, compared with other subtypes, TNBC has a unique TME, a higher immune infiltration and heterogeneity, and is characterized by the formation of pre-metastasis niche. In addition, we found that miRNAs expressed higher in the sEVs than in the cells, indicating the enrichment of miR-106b-5p and miR-18a-5p in the sEVs of BC cells (Fig. 6E-F). In conclusion, sEVs miR-106b-5p and miR-18a-5p may act as important M2 polarization regulating molecules of macrophages.

To further determine the upregulation of PD-L1 in macrophages induced by sEVs-miR-106b-5p and sEVs-miR-18a-5p, overexpression mimics were used to transfect BC cells, respectively. It was found that the transfected cells can effectively secrete high levels miR-106b-5p and miR-18a-5p sEVs (Supplementary Fig. 3D). These sEVs of the control and the overexpression groups were co-cultured with M0 cells, and it was found that the sEVs of BC cells with high levels of miR-106b-5p and miR-18a-5p significantly promote the PD-L1 expression in macrophages based on the RT-qPCR and WB results (Fig. 6G-H). Compared with macrophages co-cultured with a single miRNA secretion, the expression of CD163 and PD-L1 was significantly increased in the co-cultured group, showing the synergistic effect on promoting the M2 polarization and PD-L1 expression of macrophages.

### BC-Derived sEV-miR-106b-5p and sEV-miR-18a-5p promotes PD-L1 expression in macrophages via the PTEN/AKT and PIAS3/STAT3 pathways

**Fig. 7:**
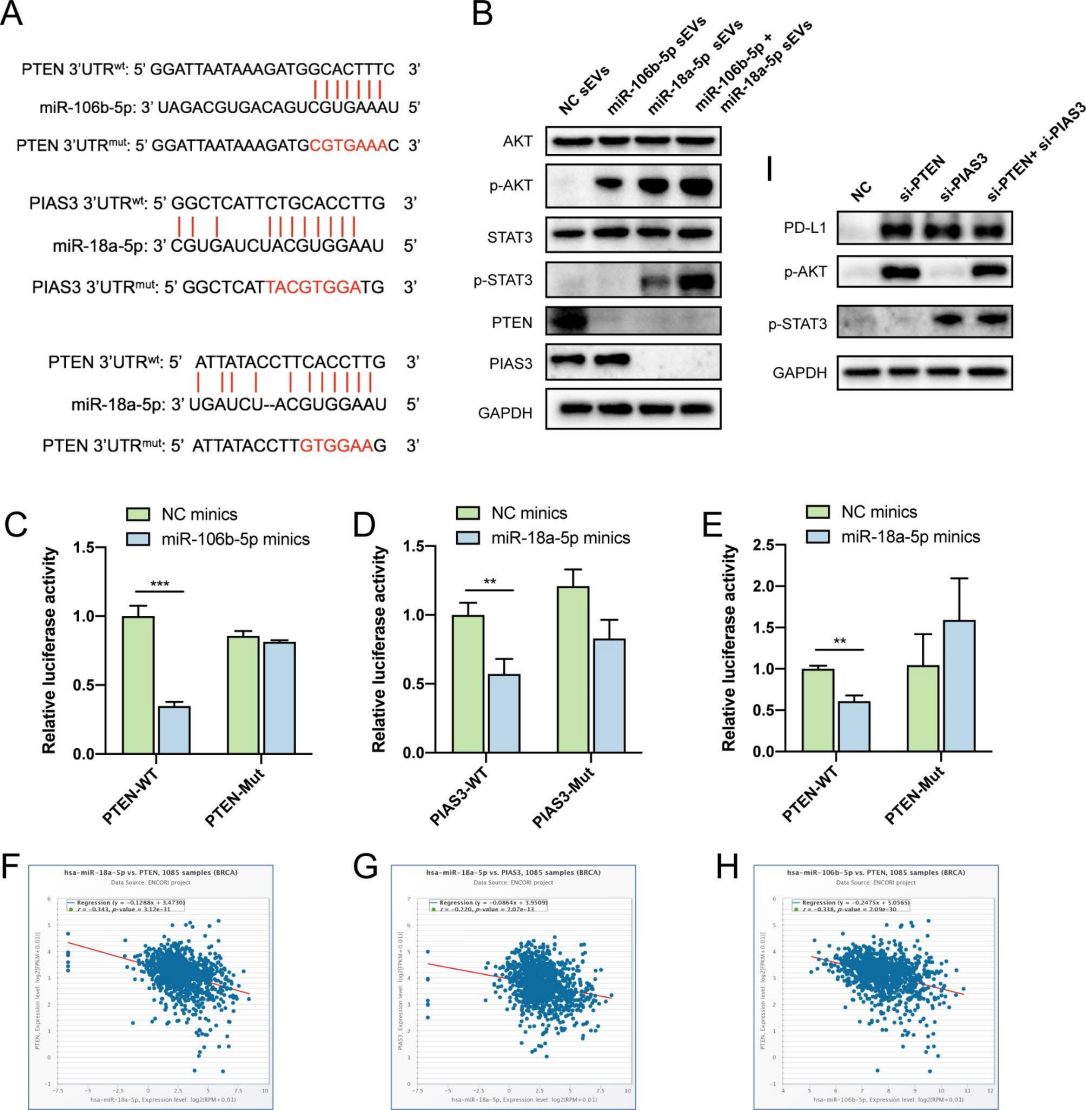
BC-derived sEV-miR-106b-5p and sEV-miR-18a-5p promoting the PD-L1 expression in macrophages via the PTEN/AKT and PIAS3/STAT3 pathways. (**A**) Potential binding sites of miR-106b-5p within the 3 ‘UTR of PTEN, and of miR-18a-5p within the 3 ‘UTR of PTEN and PIAS3. (**B**) The protein expression of PTEN/AKT and PIAS3/STAT3 pathways in macrophages co-cultured with sEVs derived by high levels miR-106b-5p and/or miR-18a-5p BC cells analyzed by WB. (**C**) Luciferase report vectors carrying PTEN-3 ‘UTR-WT and PTEN-3 ‘UTR-Mut were co-transfected with NC or miR-106b-5p mimics. Dual-luciferase reporter assays were conducted to determine luciferase activity. (**D-E**) Luciferase report vectors carrying PTEN-3 ‘UTR-WT, PTEN-3 ‘UTR-Mut, PIAS3-3 ‘UTR-WT and PIAS3-3 ‘UTR-Mut were co-transfected with NC or miR-18a-5p mimics. Dual-luciferase reporter assays were conducted to determine luciferase activity. (**F-H**) Correlation between expression of PIAS3 or PTEN and miR-106b-5p or miR-18a-5p. (**I**) The protein expression of PD-L1 in macrophages transfected with si-PTEN and si-PIAS3.

PI3K-AKT and JAK-STAT pathways are important molecular mechanisms to regulate PD-L1 expression and M2 macrophage polarization [30–32]. To investigate the underlying molecular mechanisms, the miR-106b-5p and miR-18a-5p target genes were forecasted using Targetscan and Starbase database. We showed that miR-106b-5p could effectively bind the 3 ‘UTR region of PTEN, while miR-18a-5p the 3 ‘UTR of both PTEN and PIAS3 (Fig. 7A). Further analyses showed that after co-cultured with sEVs derived by high levels of miR-106b-5p and miR-18a-5p BC, PTEN was significantly downregulated, while p-AKT increased. Also, sEV-miR-18a-5p was found able to downregulate PIAS3 in the JAK-STAT pathway and upregulate p-STAT3 in macrophages (Fig. 7B). Dual-luciferase reporter assays were conducted to determine the binding of PTEN and PIAS3 by miR-106b-5p and miR-18a-5p, respectively. The results showed the inhibiting role of miR-106b-5p on the expression of reporter vectors carrying PTEN-3 ‘UTR-WT, while reporter vectors containing PTEN-3 ‘UTR-Mut were completely unchangeable in macrophages (Fig. 7C). Likewise, miR-18a-5p was able to inhibit the expression of reporter vectors carrying PTEN-3 ‘UTR-WT and PIAS3-3 ‘UTR-WT, whereas the mutant PTEN-3 ‘UTR and PIAS3 3’UTR were not affected (Fig. 7D-E). A negative correlation between miR-106b-5p and PTEN expression in BC was evidenced, as well as a between miR-18a-5p and the expression of PIAS3 and PTEN (Fig. 7F-H).

In order to further determine whether miR-106b-5p and miR-18a-5p can upregulate PD-L1 expression through PTEN/AKT and PIAS3/STAT3 pathway, we used siRNA to silence key factor PTEN or PIAS3. The transfection efficiency was detected by RT-qPCR. Thus, we chose si-PTEN-1 and si-PIAS3-2 to conduct the next experiment (Supplementary Fig. 3E-F). WB showed that si-PTEN and si-PIAS3 can improve the PD-L1 level of macrophages, suggesting the function of PTEN/AKT and PIAS3/STAT3 pathways in upregulating PD-L1 (Fig. 7I).

## Discussion

Although the treatment of BC has made great progress in the past decades, a relevant proportion of BC patients still develop metastasis, resulting in treatment failure and death [4]. Therefore, only the elimination of tumor cells may not effectively inhibit the progress of later stages. The heterogeneity of this tumor relies on the presence of tumor cells and interstitial cells in the TME of BC [7]. Macrophages are the most abundant immune cells in the TME and were associated with a poor clinical outcome [33]. M2 macrophages can inhibit the cell-mediated immune response, leading to immune evasion, immunosuppression, and reduction of the efficacy of chemotherapy and radiotherapy [34, 35]. However, since macrophages have a functional and phenotypical plasticity, they can have different and complex functions in response to different stimulations [36]. In this study, we confirmed that TAMs were significantly higher in metastatic BC tissues than in BC tissues of earlier stages, as well as the proportion of M2 TAMs, indicating the active role of TAMs in the metastatic process, which in turn may also be stimulated by BC cells in order to polarize into M2 phenotype. However, the phenotype and complexity functions of TAMs in this regard have been poorly characterized.

PD-L1 often reported to promote immune evasion of tumor cells by exhausting T cells [17, 18]. However, previous studies mainly focused on PD-L1 expression in tumor cells and the related effects on suppressing T cells, the heterogeneity of PD-L1 expression in other different immune cells has not been investigated yet [37]. *Yin et al.* found that PD-L1 was expressed more in TAMs than in colorectal cancer cells [38], while *Ossama et al.* found that the expressions of PD-L1 was consistent in tumor and immune cells [39]. Consistently with the first study, in advanced breast cancer, we found that PD-L1 was more abundant in TAMs rather than in BC cells, and among the TME, PD-L1 was mostly expressed in macrophages, and co-located with CD163 + macrophages. Thus, we suggested that the new PD-L1 + CD163 + TAM subgroup can be considered a predictor factor of BC metastasis.

sEVs play significant roles in the intercellular communication between tumor cells and metastasis TME to form the pre-metastasis niche [40, 41], and since macrophages show more phagocytic activity than other cells, we speculated that the sEVs released by BC cells were mainly up taken by macrophages and involved in their programming. Also, several studies reported that sEVs can reflect their cell origin and disease stage by the transported bioactive substances, and thus they can serve as potential biomarkers for diagnosis and prognosis [42]. MiRNA represents a small non-coding RNA which can inhibit the translation and stability of mRNA by targeting its 3’-UTR regions [43]. MiRNAs are widely distributed in body fluids when they can be degraded by nuclease, while the lipid bilayer membrane of sEVs showed resistance to nucleases. In this study, miRNA-sequencing investigated the miRNAs expression profiles of plasma sEVs in metastasis BC patients and a subsequent validation was performed by RT-qPCR in a broad patient cohort. High levels of plasma sEV-miR-106b-5p, miR-17-5p, miR-18a-3p, miR-18a-5p, were highly predictive of metastasis and poor OS in BC patients, suggesting that plasma sEV-miRNA might be a valuable tool to be used to monitor metastatic BC cells and to predict the prognosis.

Further studies elucidated the mechanisms by which PD-L1 was upregulated in BC TAMs. We found that BC-derived sEV-miR-106b-5p and sEV-miR-18a-5p can be effectively transmitted to TAMs, inducing simultaneously PD-L1 expression in macrophages and regulate their polarization. Also, based on previous reports indicating that PI3K/AKT and JAK/STAT pathways modulated PD-L1 expression and M2 polarization [30–32], we proved that BC derived sEV miR-106b-5p and miR-18a-5p facilitate the phosphorylation of these two pathways in TAMs by inhibiting PTEN and PIAS3 3’ UTR. In addition, miR-106b promoted the progress of BC through PTEN/PI3K/Akt pathways [44]. *Shen et al* demonstrated that miR-18a may have a dual function in promoting or inhibiting the development of human cancers, in relation to different stages or subtypes of cancer, even in the same organ [45]. From the findings of this study, we concluded that key signaling functions of sEV miR-106b-5p and miR-18a-5p are the regulation of PD-L1 expression in macrophages and regulate their polarization.

At present, increased studies focused on the function of PDL1 on macrophages. It has been reported that blocking the expression of PD-L1 in macrophages with PD-L1 antibody can promote the proliferation of macrophages and the differentiation of inflammatory phenotype [46]. Also, *Lu et al.* suggested that PD-L1 signals concealed the intrinsic anti-tumor properties of macrophages which was controlled by glycolytic metabolism [47]. In the future study, we will focus on the role and mechanism of PD-L1 in macrophage polarization in BC, thus establishing combination therapy between PD-L1 checkpoint blockade therapy and macrophage phenotype modulators.

## Conclusions

**Fig. 8:**
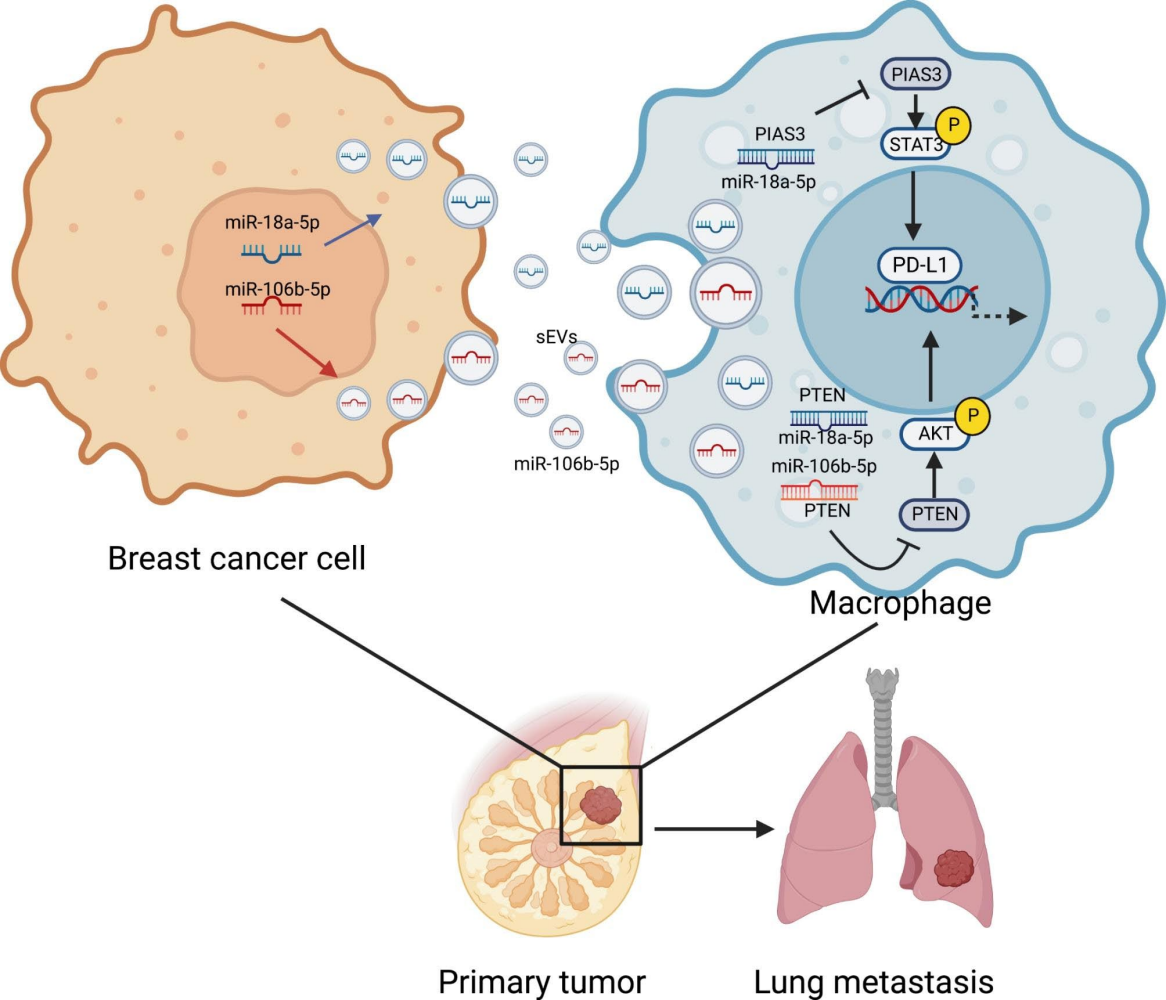
Schematic illustration of BC-derived sEVs enhance PD-L1 expression in M2 polarization TAMs by regulating miR-106b-5p/PTEN/AKT/PD-L1 and miR-18a-5p/PIAS3/STAT3/PD-L1 signaling pathways, thus promote BC tumor progression

In conclusion, our study demonstrated a specific PD-L1 + CD163 + macrophage subgroup abundant in BC, and we clarified the function of macrophages in metastatic BC. It was found that BC cells transmit sEV-miR-106b-5p and sEV-miR-18a-5p to macrophages and induce the PD-L1 expression through PTEN/AKT and PIAS3/STAT3 signaling pathways, which led to the macrophages polarization and development of BC metastasis (Fig. 8). Therefore, the inhibition of the specific interactions of miR-106b-5p/PTEN/AKT/PD-L1 and miR-18a-5p/PIAS3/STAT3/PD-L1 signaling pathways is a crucial step that represent a promising target for future therapeutic strategies for treatment of BC.

## Electronic supplementary material

Below is the link to the electronic supplementary material.

Supplementary Material 1

Supplementary Material 2

## Data Availability

The data underlying this article will be shared on reasonable request to the corresponding author.
