# Supplementary material for: Tumor-derived small extracellular vesicles promote breast cancer progression by upregulating PD-L1 expression in macrophages
Source: Cancer Cell Int. 2023 Jul 14;23:137. doi: 10.1186/s12935-023-02980-0 (PMC10347751; doi:10.1186/s12935-023-02980-0)
Supplement: Supplementary file 1 — Supplementary Material 1 [file 12935_2023_2980_MOESM1_ESM.docx]

**Supplementary Table 1 Clinical characteristic of breast cancer patients**

| **Variables** | **Normal group（N=20）** | **Early BC group（N=20）** | **Advanced BC group（N=20）** | **p** |
| --- | --- | --- | --- | --- |
| **Age** | 43±8.86 | 50±7.70 | 52±14.17 | 0.138 |
| **Tumor size** | / | 1.46±0.44 | 3.13±1.82 | **0.013** |
| **T stage** |  |  |  | **0.023** |
| 1 | / | 20 | 8 |  |
| 2 | / | 0 | 8 |  |
| 3-4 | / | 0 | 6 |  |
| **N stage** |  |  |  | **0.001** |
| 0 | / | 20 | 0 |  |
| 1 | / | 0 | 2 |  |
| 2 | / | 0 | 10 |  |
| 3 | / | 0 | 10 |  |
| **M stage** | 0 | 0 | 20 | **0.001** |
| **Pathological stage** |  |  |  | **0.001** |
| I- II | / | 20 | 0 |  |
| III-IV | / | 0 | 20 |  |
| **ki67 status** | / | 15.36±10.85 | 49.55±16.50 | **0.001** |

T, primary tumor; N, regional lymph nodes; M, distant metastasis

**Supplement table 2. List of antibodies**

| Antigens | Manufacturer | Application |
| --- | --- | --- |
| Calnexin | Abcam，ab92573 | 1:1000 for WB |
| CD9 | Abcam，ab236630 | 1:1000 for WB |
| TSG101 | Abcam，ab125011 | 1:1000 for WB |
| CD68 | Abcam，ab213363 | 1:3000 for WB, 1:2000 for IHC and IF |
| CD163 | Abcam，ab182422 | 1:1000 for WB, IHC and IF |
| PD-L1 | CST, 13684 | 1:1000 for WB, 1:500 for IHC IF |
| Akt | CST, 4685 | 1:1000 for WB |
| Phospho-Akt (Ser473) | CST, 4060 | 1:1000 for WB |
| Stat3 | CST, 9139 | 1:1000 for WB |
| Phospho-Stat3 (Tyr705) | CST, 9145 | 1:1000 for WB |
| PTEN | CST, 9188 | 1:1000 for WB |
| PIAS3 | CST, 9042 | 1:1000 for WB |
| GAPDH | CST, 92310 | 1:3000 for WB |

**Supplement table 3. Sequences of primers for miRNA First Strand cDNA Synthesis**

| miRNA ID | Primer（5' to 3'） |
| --- | --- |
| hsa-miR-362-5p | CGAATCCTTGGAACCTAGGTGTGAG |
| hsa-miR-190a-5p | GCGCGCGCGTGATATGTTTGATATAT |
| hsa-miR-144-5p | CGCGCGCGGATATCATCATATACTGTA |
| hsa-miR-124-3p | TAAGGCACGCGGTGAATGC |
| hsa-miR-576-5p | GCGCGATTCTAATTTCTCCACGTCTTT |
| hsa-miR-183-5p | TCGCTATGGCACTGGTAGAATTCACT |
| hsa-miR-182-5p | CGCTTTGGCAATGGTAGAACTCACA |
| hsa-miR-501-5p | CAATCCTTTGTCCCTGGGTGAGA |
| hsa-miR-93-5p | TCAAAGTGCTGTTCGTGCAGGTAG |
| hsa-miR-17-5p | TGCAAAGTGCTTACAGTGCAGGTAG |
| hsa-mir-106a | TGCAAAGTGCTTACAGTGCAGGTAG |
| hsa-miR-18a-5p | GCGTAAGGTGCATCTAGTGCAGATAGA |
| hsa-miR-20b-5p | TGCAAAGTGCTCATAGTGCAGGTAG |
| hsa-miR-144-3p | CCGCGCGTACAGTATAGATGATGTACT |
| hsa-miR-548ae-5p | GCGCGCGAAAAGTAATTGTGGTTTTTG |
| hsa-miR-106b-5p | CGCTAAAGTGCTGACAGTGCAGAT |
| hsa-miR-548au-5p | CGCGCGAAAAGTAATTGCGGTTTTTG |
| hsa-miR-548o-5p | CGCGCGAAAAGTAATTGCGGTTTTTG |
| hsa-miR-18a-3p | ACTGCCCTAAGTGCTCCTTCTGA |
| hsa-miR-5001-3p | TTCTGCCTCTGTCCAGGTCCT |
| hsa-miR-624-5p | CGCGTTTGGCACTAGCACATTTTTG |
| hsa-miR-96-5p | CGCGTTTGGCACTAGCACATTTTTG |
| hsa-miR-548bc | GCGCGCAAAAACTGTGATTACTTTTGC |
| hsa-miR-7-5p | CGCGTGGAAGACTAGTGATTTTGTTGT |
| cel-miR-39-3p | TCACCGGGTGTAAATCAGCTTG |

**Supplement table 4 Sequences of forward primers and reverse primers using for RT-qPCR**

| mRNA/miRNA ID | | Primers（5' to 3'） | |
| --- | --- | --- | --- |
| PD-L1 forward | | TGGCATTTGCTGAACGCATTT | |
| PD-L1 reverse | | TGCAGCCAGGTCTAATTGTTTT | |
| ARG1 forward | | GTGGAAACTTGCATGGACAAC | |
| ARG1 reverse | | AATCCTGGCACATCGGGAATC | |
| CD206 forward | | TCCGGGTGCTGTTCTCCTA | |
| CD206 reverse | | CCAGTCTGTTTTTGATGGCACT | |
| IL10 forward | | GACTTTAAGGGTTACCTGGGTTG | |
| IL10 reverse | | TCACATGCGCCTTGATGTCTG | |
| GAPDH forward | | GGAGCGAGATCCCTCCAAAAT | |
| GAPDH reverse | | GGCTGTTGTCATACTTCTCATGG | |
| hsa-miR-106b-5p forward | GCTGCGTAAAGTGCTGACAGT | |  |
| hsa-miR-106b-5p reverse | AGTGCAGGGTCCGAGGTATT | |  |
| hsa-miR-106b-5p RT primer | GTCGTATCCAGTGCAGGGTCCGAGGTATTCGCACTGGATACGACATCTGC | |  |
| hsa-miR-18a-5p forward | GCGTAAGGTGCATCTAGTGCA | |  |
| hsa-miR-18a-5p reverse | AGTGCAGGGTCCGAGGTATT | |  |
| hsa-miR-18a-5p RT primer | GTCGTATCCAGTGCAGGGTCCGAGGTATTCGCACTGGATACGACTCTATC | |  |
| hsa-miR-362-5p forward | GCGAATCCTTGGAACCTAGGT | |  |
| hsa-miR-362-5p reverse | AGTGCAGGGTCCGAGGTATT | |  |
| hsa-miR-362-5p RT primer | GTCGTATCCAGTGCAGGGTCCGAGGTATTCGCACTGGATACGACACTCAC | |  |
| hsa-miR-501-5p forward | GCGAATCCTTTGTCCCTGG | |  |
| hsa-miR-501-5p reverse | AGTGCAGGGTCCGAGGTATT | |  |
| hsa-miR-501-5p RT primer | GTCGTATCCAGTGCAGGGTCCGAGGTATTCGCACTGGATACGACTCTCAC | |  |
| U6 forward | CTCGCTTCGGCAGCACA | |  |
| U6 reverse | AACGCTTCACGAATTTGCGT | |  |
