# Supplementary material for: Tumor-derived small extracellular vesicles promote breast cancer progression by upregulating PD-L1 expression in macrophages
Source: Cancer Cell Int. 2023 Jul 14;23:137. doi: 10.1186/s12935-023-02980-0 (PMC10347751; doi:10.1186/s12935-023-02980-0)
Supplement: Supplementary file 2 — Supplementary Material 2 [file 12935_2023_2980_MOESM2_ESM.docx]

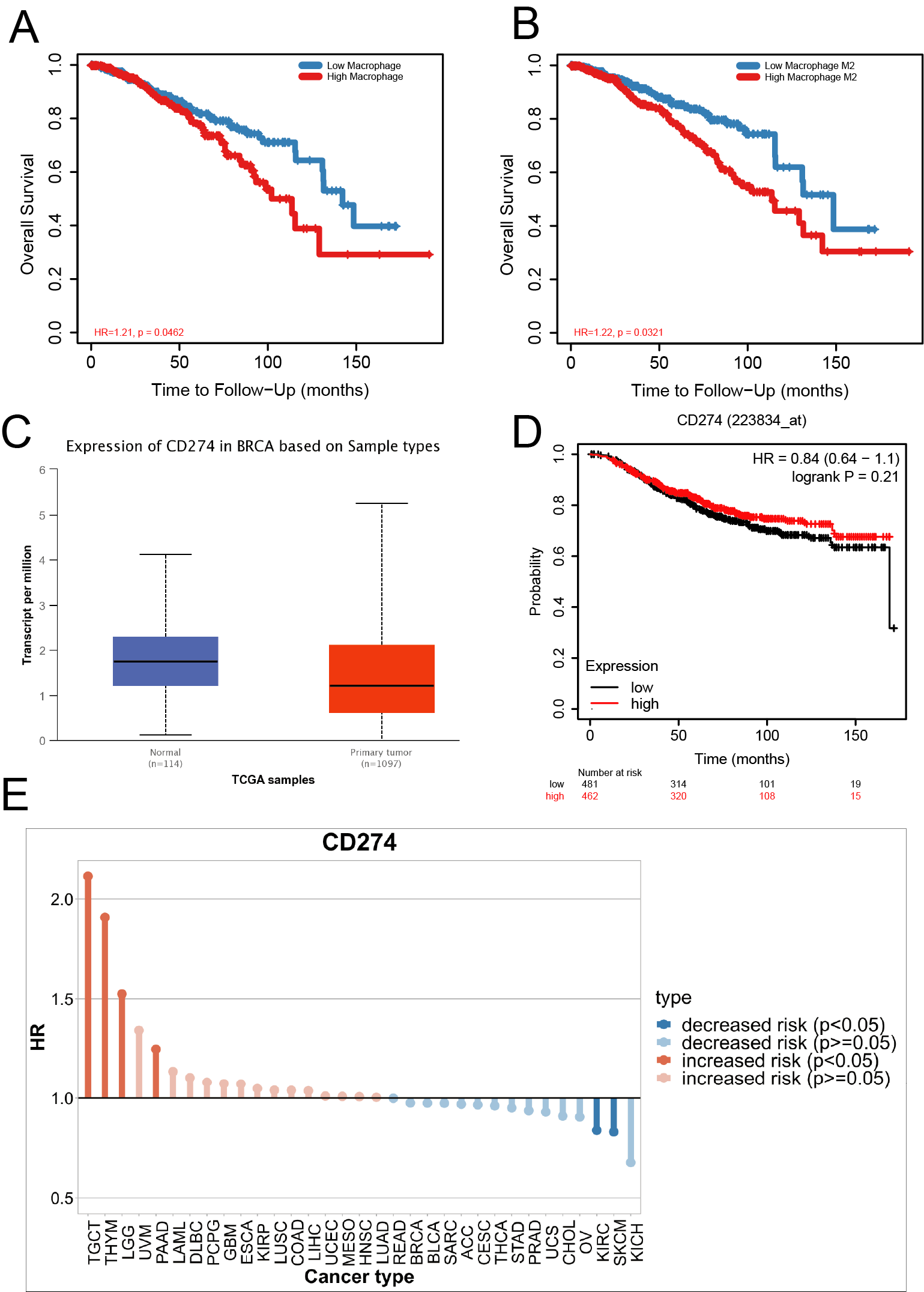


Supplementary Figure 1. (A-B) Overall survival analysis of macrophages and M2 type macrophages infiltrate in breast cancer. (C) Different expression of PD-L1 between invasive breast cancer and normal breast tissue by TCGA database. (D) Kaplan–Meier curves showing overall survival of PD-L1 in breast cancer by TCGA database. (E) Kaplan–Meier curves showing overall survival of PD-L1 in different types of cancer by TCGA database.


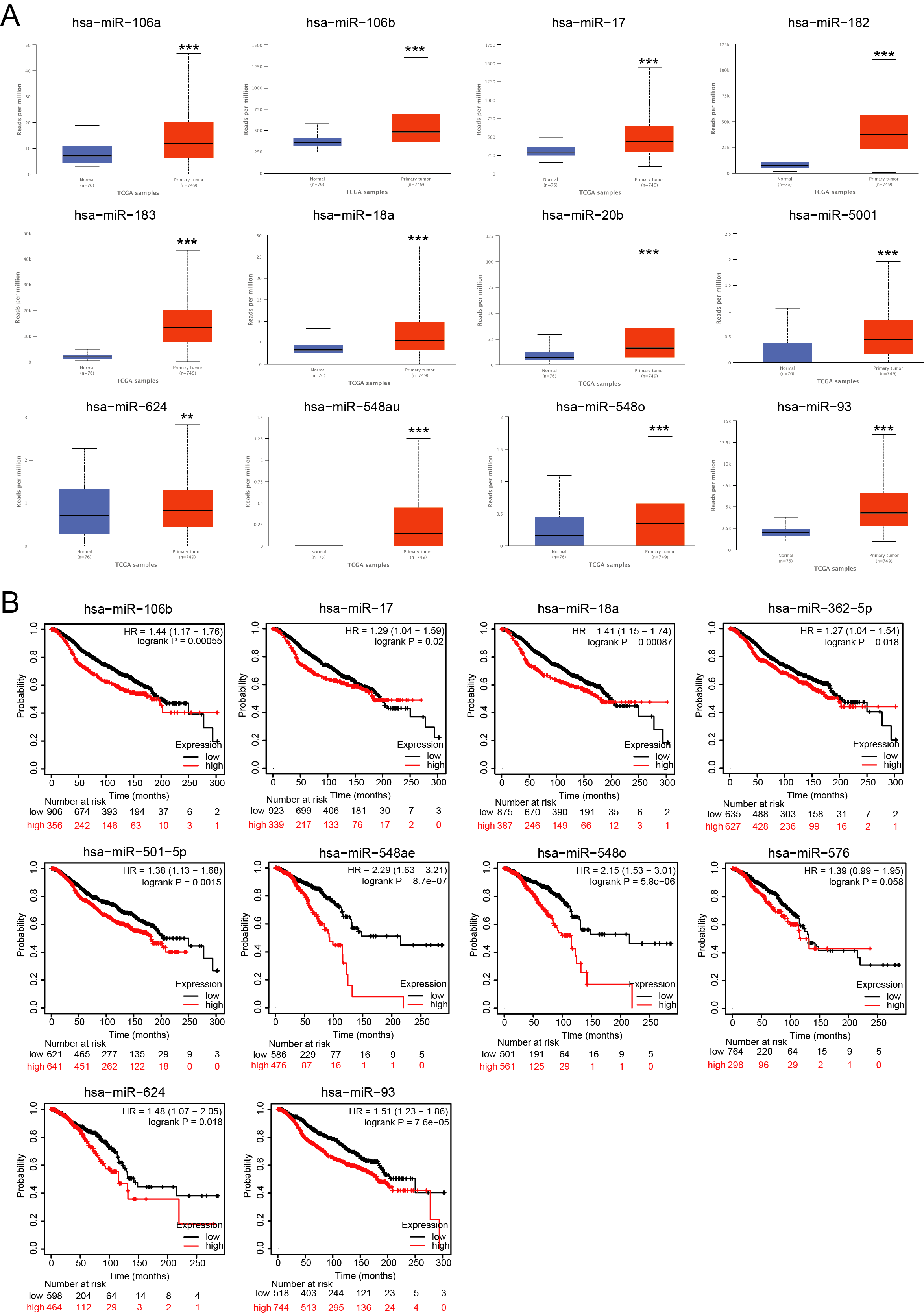


Supplementary Figure 2. (A) Expression of differentially expressed miRNAs between invasive breast cancer and normal breast tissue by TCGA database. (B) Kaplan–Meier curves showing overall survival analysis of differentially expressed miRNAs in breast cancer by TCGA database.


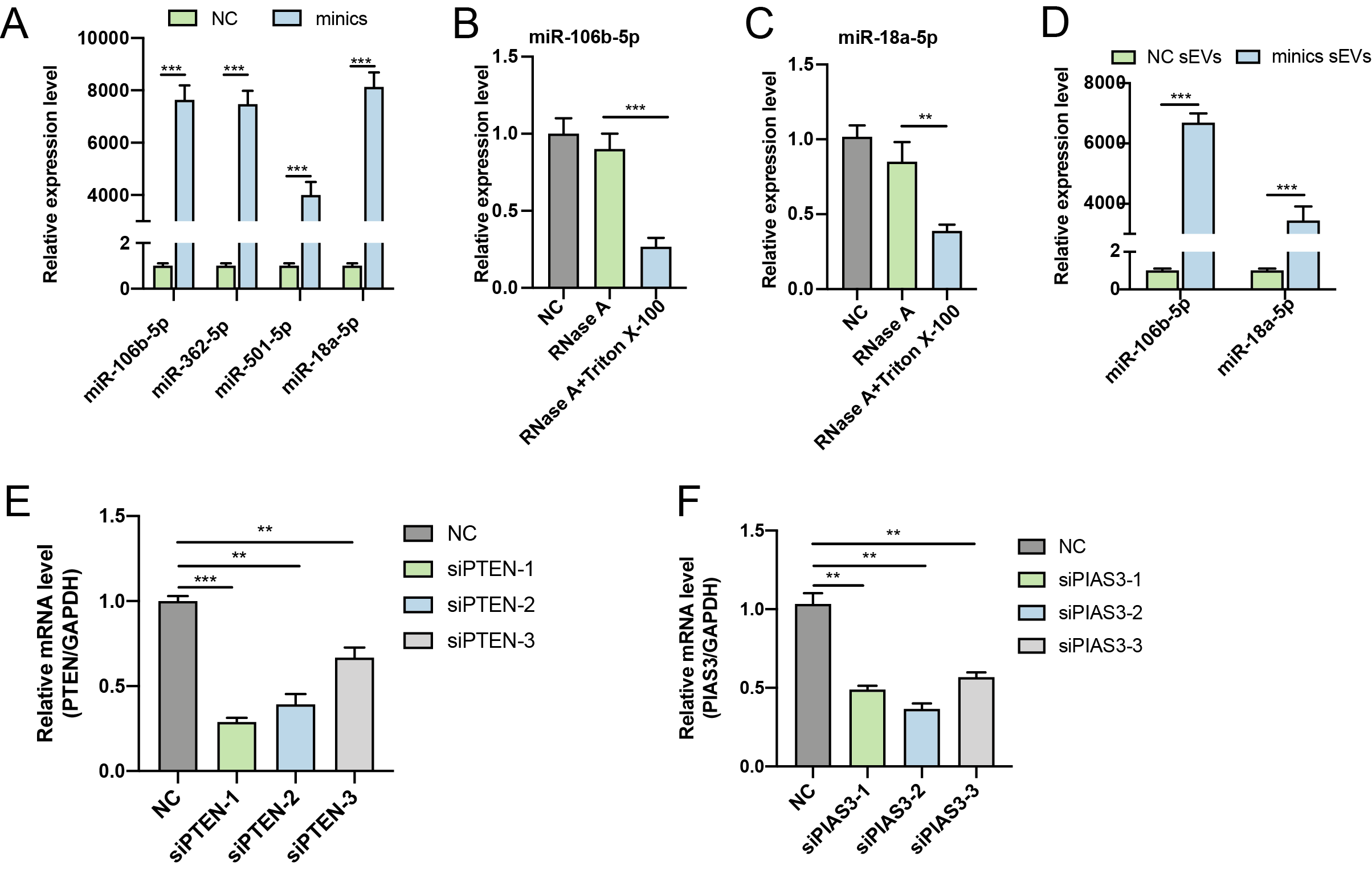


Supplementary Figure 3. (A) The transfection efficiency of miR-106b-5p, miR-18a-5p, miR-501-5p and miR-362-5p minics detected by RT-qPCR. (B-C) The levels of miR-106b-5p and miR-18a-5p in MDA-MB-231 cell culture medium before and after treatment with RNase A and Triton X-100 by RT-qPCR. (D) The differential expression of miR-106b-5p and miR-18a-5p in breast cancer cells and sEVs by RT-qPCR. (E) The transfection efficiency of si-PTEN was detected by RT-qPCR. (F) The transfection efficiency of si-PIAS3 was detected by RT-qPCR.
